# THE COST OF IMBALANCE: HOW BALANCE PROBLEMS DRIVE ENERGY COST OF WALKING IN PERSONS WITH MULTIPLE SCLEROSIS

**DOI:** 10.2340/jrm.v58.45814

**Published:** 2026-09-18

**Authors:** Sjoerd T. TIMMERMANS, Arianne S. GRAVESTEIJN, Koen WISHAUPT, Heleen BECKERMAN, Marjolein M. VAN DER KROGT, Vincent DE GROOT

**Affiliations:** 1 Amsterdam UMC location Vrije Universiteit Amsterdam, Rehabilitation Medicine, Amsterdam, the Netherlands; 2 MS Center Amsterdam, Amsterdam UMC location Vrije Universiteit, Amsterdam, The Netherlands; 3 Amsterdam Movement Sciences, Rehabilitation & Development, Amsterdam, The Netherlands; 4 Amsterdam Neuroscience, Neuroinfection and Inflammation Amsterdam, The Netherlands; 5 Amsterdam Movement Sciences, Ageing & Vitality, Amsterdam, The Netherlands; 6 Maastricht University, NUTRIM School of Nutrition and Translational Research in Metabolism, Department of Nutrition and Movement Sciences, Maastricht, The Netherlands

**Keywords:** multiple sclerosis, oxygen consumption, postural balance, walking speed

## Abstract

**Introduction:**

People with multiple sclerosis have difficulties walking and show a high energy cost of walking. Balance problems may contribute.

**Aim:**

To investigate the relationship between dynamic balance and energy cost of walking in people with multiple sclerosis, and whether walking speed affects this relationship.

**Methods:**

In a cross-sectional cohort, 20 people with multiple sclerosis walked on an instrumented treadmill at a comfortable and fast walking speed. The energy cost of walking was derived from oxygen consumption. Dynamic balance was approximated as the medio-lateral margin of stability and using balance board testing. Linear mixed models were used, with walking speed as effect modifier.

**Results:**

The mean energy cost of walking was 4.99 J/kg/m at a comfortable and 4.84 J/kg/m at a fast walking speed. The medio-lateral margin of stability was significantly associated with energy cost of walking, with walking speed a significant effect modifier (β = –27.07, 95% CI: –49.32; –4.83). At lower speeds this association was stronger, attenuating at higher speeds.

**Discussion:**

Poorer dynamic balance was associated with higher energy cost of walking, predominantly at lower walking speeds. Jointly improving walking speed and dynamic balance may reduce the energetic burden of walking in this population.

## LAY ABSTRACT

People with multiple sclerosis often find walking difficult and tiring. One reason may be that balance problems force them to walk in a less efficient way. In this study, 20 people with multiple sclerosis walked on a treadmill at 2 speeds – their normal comfortable pace and a faster pace – while we measured how much energy they used and how well they maintained their balance. We found that people who struggled more with sideways balance used more energy when walking, but, importantly, this was mainly the case at slower walking speeds. At faster speeds, the link between balance and energy use was weaker. This suggests that helping people with multiple sclerosis to walk faster and improve their balance at the same time – rather than focusing on one alone – may be the most effective way to make walking less tiring.

The majority of people living with multiple sclerosis (PwMS) experience walking difficulties, which hinder daily activities, reduce societal participation, and negatively impact quality of life (1). The energy cost of walking (ECw) can be a valid marker of walking difficulties (2). In healthy people, the ECw is closely related to walking speed, forming a U-shaped curve with habitual walking speed at the lowest point of this curve (3). Several factors may increase ECw in PwMS (4–6), including altered spatiotemporal gait characteristics, such as reduced walking speed (4), decreased stride (4) and step length (7), prolonged double limb support (4, 8), and increased step width (7), which leads to a leftwards and upwards shift on the U-shaped curve (9). PwMS can expend up to almost 3 times more energy during walking compared with healthy controls (2). The role of different motor impairments underlying the increased ECw in PwMS is not fully understood (10). Among paresis, coordination problems, and spasticity, another potential contributing factor could be impaired balance, resulting in a less efficient gait pattern with compensatory movements and higher ECw (11). Because of the complex non-linear relations and interactions between these factors, it can be difficult to tailor rehabilitation treatment.

Balance problems are prevalent in PwMS and can be categorized into static balance (i.e., maintaining balance during stance) and dynamic balance (i.e., maintaining balance while walking or doing activities) problems (8, 12). Dynamic balance can be approximated using the margin of stability (MoS). The MoS represents the distance between the body’s centre of mass (CoM) and the boundary of the base of support (BoS), which is determined by foot placement. It incorporates both the position and velocity of the CoM, as well as step width and step length, enabling a comprehensive assessment of mediolateral and anteroposterior stability during walking (13). A larger mediolateral (ML) MoS reflects a balance control strategy involving wider steps that mechanically increase base-of-support stability, whereas a smaller ML MoS indicates that the person maintains lateral stability with a narrower step, reflecting more efficient balance control during gait (14, 15). The ML MoS is determined by the distance between the extrapolated CoM and the lateral border of the foot, whereby wider steps result in a greater ML MoS and narrower steps in a smaller ML MoS (14). In PwMS, a larger ML MoS may therefore reflect a compensatory wide-step strategy rather than true lateral stability, as walking more slowly with wider steps can increase ML MoS despite an elevated fall risk (15, 16). An alternative for dynamic balance assessment in daily clinical practice is a balance board test, which challenges dynamic balance through tandem gait and has been shown to detect subtle balance disturbances in PwMS that may not be apparent on standard clinical assessments (12). While the ML MoS and the balance board test both aim to capture dynamic balance, they assess different aspects: ML MoS provides a continuous, biomechanical measure of mediolateral stability during walking, whereas the balance board offers a discrete, clinically accessible index of balance impairment. By examining both measurement methods, it can be determined whether laboratory-based and clinical assessment of dynamic balance are similarly related to ECw. As dynamic balance plays an important role in overall mobility and risk of falls, it makes the evaluation and treatment of balance problems clinically relevant (11).

Despite these findings, the extent to which balance problems contribute to the elevated ECw in PwMS remains unclear. Both dynamic balance and ECw may be affected by walking speed, complicating their relationship (16). In PwMS, a lower walking speed can lead to a higher ECw (2, 4, 17, 18). There is conflicting evidence on how walking speed impacts dynamic balance in PwMS, as studies have shown that increasing walking speed might improve dynamic balance (19), but decreasing walking speed might also serve as a compensatory strategy to minimize the influence of balance disturbances (17). PwMS might reduce walking speed and step length and increase step width to reduce the lateral momentum of the body, making it easier to keep the extrapolated CoM within the base of support (17).

To tailor rehabilitation strategies targeting walking difficulties in PwMS, it is essential to understand factors contributing to walking dysfunction and their complex interrelationships. Therefore, the objectives of this study were (*i*) to investigate the relationship between dynamic balance and ECw, as quantified by the ML MoS and the clinically applicable balance board, in PwMS, and (*ii*) to investigate the role of walking speed in the relationship between dynamic balance and ECw. It was hypothesized that balance problems are associated with higher ECw at comfortable walking speed, and that this relationship decreases at fast walking speed, leading to a decrease in MoS.

## METHODS

### Design and participants

In this cross-sectional study we included adult PwMS with walking problems referred to the rehabilitation medicine outpatient clinic of the Amsterdam UMC, location VUmc between January 2021 and February 2023. Other inclusion criteria were being able to walk for 6 min on a motorized treadmill, and being relapse free in the previous 30 days. PwMS were excluded when they were fully dependent on assistive devices for walking or had orthopaedic or traumatological injury to the legs that influenced their current walking ability. All participants provided written informed consent prior to participation. Medical ethical approval was waived by the medical ethical review board of the VU university medical centre, Amsterdam (METc 2020.461).

### Procedures

During a 3-h session, participants underwent a balance test and prolonged walking trials at comfortable (CWS) and fast walking speed (FWS) and ECw was measured. The session began with the assessment of patient anthropometry, including body height, weight, and leg length. The legs were categorized as most impaired leg (MIL) and least impaired leg (LIL) based on paresis and sensibility testing during physical examination and self-report. Prior to clinical measurements participants completed the MS Walking Scale-12 questionnaire (20) digitally using Castor EDC (2019) (https://www.castoredc.com/).

PwMS performed the balance board test, a clinical assessment of dynamic balance (12). During this test, participants walked in tandem gait with arms crossed in front of their chest, first on a 10-foot taped line and then on a 10-foot balance board (0.75 inches high and 4.5 inches wide) (12), both divided into quarters. To complete the taped line test and move onto the balance board, participants had to pass at least one-quarter of the tape without stepping aside or uncrossing their arms. The score was determined by the number of quarters successfully completed after 3 trials on both the taped line and balance board, with a maximum of 24 and a minimum of 0 quarters. The final score was calculated as percentage of completed quarters (total quarters/24).

Subsequently, several introductory walking trials were performed. Participants familiarized themselves with treadmill walking on the Gait Realtime Analysis Interactive Lab (GRAIL, Motekforce Link B.V., Amsterdam, The Netherlands) for 10 min, to prevent habituation effects. To determine comfortable walking speed (CWS), participants completed an overground 6-minute walk test (6MWT) at an oval track of 34 or 30 m (based on availability). PwMS were instructed to walk at a pace that was easy to maintain and comfortable for 6 min. To determine fast walking speed (FWS), participants were then encouraged to walk at the fastest speed they should be able to maintain for 6 min, completing 2 laps. Breath-by-breath oxygen consumption (VO_2_) was measured during 5 min of supine rest, during overground walking, and during walking on the GRAIL, using a wearable metabolic system (K5, COSMED, Rome, Italy).

Prior to treadmill walking, 26 passive retroreflective skin-mounted markers were placed on participants, corresponding with the Human Body Model (HBM) (21), excluding the head and arms. At the dual-belt instrumented treadmill, PwMS walked for 6 min at their overground CWS, followed by 6 min at their overground FWS. All participants wore a safety harness during the treadmill trials. A 10-camera motion capture system at 100 Hz (10 Bonitas, Vicon Motion Systems Ltd, Oxford, UK) was used to record the 3-dimensional marker coordinates. Force sensors underneath both treadmill belts (50x200cm) measured the ground reaction forces.

### Data analysis

**Fig 1.**
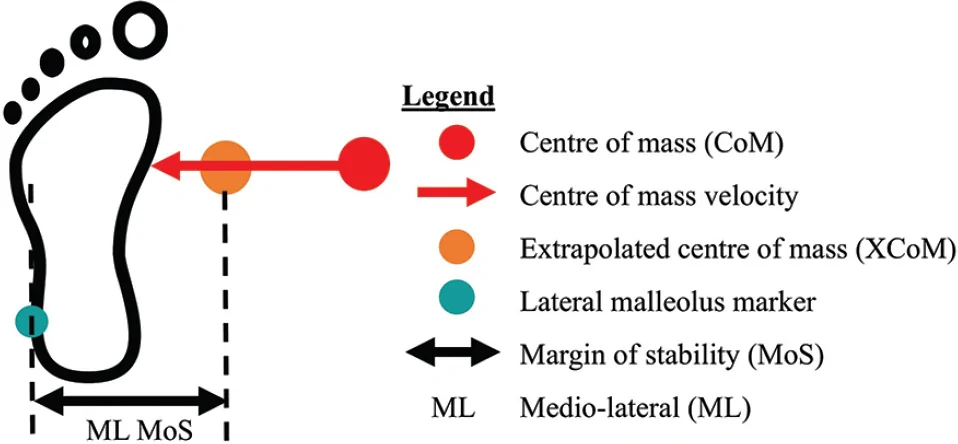
Medio-lateral margin of stability.

ML MoS was calculated during the first and last minute of both the CWS and FWS trial, adapted from the method described by Hof et al., “MoS = BoS – XCoM” (2005) (13), and as used by Peebles at al. (16). Here, BoS is the location of the boundary of the Base of Support (BoS), defined by the markers on the lateral malleoli, and XCoM is the extrapolated centre of mass (CoM). The CoM was estimated as the mean position of the 4 pelvic markers. CoM velocity was obtained by numerical differentiation of the CoM position signal. The XCoM was then calculated as XCoM = CoM + (v_CoM/ω_0_), where ω_0_ = √(g/L), g = 9.81 m/s², and l was approximated as the maximum vertical position of the CoM above the ground during the trial (13) (Fig. 1). ML MoS was calculated at the instant of ipsilateral heel contact for each stride, defined as the first sample of each kinematic gait cycle. The mean ML MoS across all strides was used for statistical analysis, consistent with the approach described by Peebles et al. (16). MoS was calculated for the most impaired leg (MIL) and least impaired leg (LIL), as side asymmetries are often seen in PwMS (22, 23). As secondary outcome measures, spatiotemporal parameters related to balance (step width, step and stride length, and double limb support) were calculated for both CWS and FWS trials.

Energy expenditure (J/kg/min) was calculated using the average interpolated V̇O_2_ in mL/kg/min data during the last 3 min of treadmill walking at CWS and FWS. V̇O_2_ data were transformed to energy expenditure in J/kg/min using Lusk’s equation (Lusk, 1924) = (15,962 + 5,155 * RER * (V̇O_2_ /1000)) and subsequently presented per walking distance (ECw = energy expenditure in J/kg/min/(walking speed in m/s * 60)) (24).

### Statistics

Differences in spatiotemporal parameters, ML MoS, and ECw between CWS and FWS, were assessed using a paired samples *t*-test for normally distributed data or a Wilcoxon signed-rank test for non-normally distributed data. Normal distribution of the data was determined by visual inspection of histograms and a Shapiro–Wilk test. To compare the 2 measures of dynamic balance a Spearman’s correlation coefficient between ML MoS and the balance board score was calculated.

The relationship between independent variable ML MoS and the dependent variable ECw was investigated using linear mixed models (LMM) for both the MIL and LIL. A random intercept was added to account for between-subject variability in ECw. The walking speed (m/s) at each measurement trial (i.e., the treadmill speed corresponding to each participant’s individually determined CWS or FWS) was added to the model to investigate whether it acted as a confounder (defined as a change of >10% in the β coefficient of ML MoS) or effect modifier (defined as a statistically significant interaction term) of the relationship between ML MoS and ECw. Walking condition (comfortable or fast) as a dichotomous variable was additionally included as an interaction term, to investigate whether the categorical contrast in walking speed had an additional effect on this relationship. Effect modification and confounding were treated as mutually exclusive interpretations of a variable’s role; if effect modification was identified, confounding was not subsequently evaluated for that variable. When a significant interaction term was present, interpretation shifted from individual coefficients to the overall model and its conditional effects, as main effects can no longer be interpreted in isolation from the moderator. All terms involved in a significant interaction were retained in the model regardless of their individual significance, as removal of non-significant terms within an interaction would misrepresent the conditional structure of the model. The LMM analyses were repeated with the balance board score as the main determinant.

All statistical analyses were conducted using IBM SPSS statistics, version 28 (IBM Corp, Armonk, NY, USA). *p*-values < 0.05 were considered statistically significant (and *p*-values < 0.10 as a trend).

## RESULTS

### Participants

A total of 26 PwMS participated in this cross-sectional study. Complete data were available for 20 PwMS (Table I). ECw could not be determined for 1 participant due to anxiety in respect of the oxygen mask. ML MoS and spatiotemporal parameters could not be validly assessed in 5 participants because they held onto the handrail for support during the trials on the GRAIL. One person did not complete the FWS trial, but was included for CWS. Most participants had relapsing-remitting MS (65%) and self-reported minimal to mild gait disability on the MSWS-12 (median score 47.9 out of maximum 60) (20).

**Table I:** Participant characteristics of patients with complete comfortable walking speed data

Demographics	Participants (*n* = 20)
Age (years), mean (SD)	49.1 (10.8)
Female sex, *n* (%)	14 (70%)
Height (cm), mean (SD)	174 (8)
Weight (kg), median [IQR]	75.0 [65.7; 80.6]
BMI (kg*m^2^), median [IQR]	23.6 [22.0; 25.6]
MS type, *n* (%)	
Relapse-remitting	13 (65%)
Secondary progressive	3 (15%)
Primary progressive	4 (20%)
EDSS score, median [IQR]	3.3 [2.0; 3.5]
MSWS-12, median [IQR]	47.9 [25.0; 54.2]
Balance characteristics	
Quarters of balance board completed (%), median [IQR]	60.4 [14.6; 99.0]

IQR: interquartile range; BMI: body mass index; MS: multiple sclerosis; EDSS: Expanded Disability Status Scale; MSWS-12: MS Walking Scale-12.

### Dynamic balance

Balance assessed with the balance board resulted in a wide range of completed quarters. Median percentage of completed quarters of the balance board was 60.4 [IQR: 14.6; 99.0]. Two patients were unable to complete any quarters, and 5 patients completed all quarters.

ML MoS did not change significantly between CWS and FWS ((95% CI [–0.001; 0.005]) with values of 0.105 (0.024) m at CWS and 0.101 (0.026) m at FWS for the MIL condition (Table II).

**Table II:** Gait characteristics of 20 people with MS during CWS and FWS treadmill trials

Characteristic		CWS *n* = 20	FWS *n* = 19	Differences CWS– FWS (in 95% CI or *p*-value for non-parametric test) *n* = 19
Energy cost and expenditure of walking				
Energy cost (J/kg/m)		4.99 (0.70)	4.84 (0.55)	0.02 (–0.17; 0.20)
Oxygen cost (mL/kg/m)		0.24 (0.03)	0.24 (0.03)	0.01 (–0.004; 0.02)
Energy expenditure (J/kg/min)		347.14 (59.30)	414.79 (85.63)	**–67.46 (–89.81; –45.11)**
Oxygen expenditure (mL/kg/min)		17.29 (3.01)	20.23 (4.15)	**–2.94 (–4.03; –1.85)**
Spatiotemporal gait parameters				
Walking speed (m/s)		1.20 (0.21)	1.41 (0.28)	**–0.22 (–0.27; –0.17)**
Cadence (steps/min)	MIL	108 [99; 113]	111 [104; 123]	**< 0.01**
	LIL	110 [104; 114]	117 [114; 125]	**< 0.01**
Double support (%)	MIL	32.2 [31.4; 33.5]	30.1 [27.5; 32.6]	**< 0.01**
	LIL	32.2 [31.5; 32.8]	30.3 [27.8; 32.0]	**< 0.01**
Step width [m]	MIL	0.14 (0.03)	0.14 (0.03)	0.001 (–0.005; 0.007)
	LIL	0.14 (0.03)	0.14 (0.03)	0.000 (–0.006; 0.006)
Step length [m]	MIL	0.58 [0.42; 0.69]	0.64 [0.53; 0.76]	**< 0.01**
	LIL	0.56 [0.41; 0.69]	0.66 [0.54; 0.74]	**< 0.01**
Stride length [m]	MIL	1.13 [0.80; 1.40]	1.28 [1.06; 1.50]	**< 0.01**
	LIL	1.11 [0.80; 1.36]	1.28 [1.05; 1.48]	**< 0.01**
Mediolateral margin of stability				
ML MoS [m]	MIL	0.103 (0.023)	0.102 (0.024)	0.001 (–0.001; 0.003)
Start (1st min)	LIL	0.097 (0.014)	0.096 (0.015)	0.002 (–0.001; 0.004)
ML MoS [m]	MIL	0.105 (0.024)	0.101 (0.026)	0.002 (–0.001; 0.005)
End (6th min)	LIL	0.099 (0.015)	0.095 (0.018)	0.003 (–0.000; 0.006)

Normally distributed data are presented as mean (SD) with paired mean differences; non-normally distributed data are presented as median [IQR] with the *p*-value of the Wilcoxon signed ranked test. 95% CI: 95% confidence interval; CWS: comfortable walking speed; ECw: energy cost of walking; FWS: fast walking speed; J/kg/m: joules per kilogram bodyweight per metre; LIL: least impaired leg; m: metre; m/s: metres per second; MIL: most impaired leg; ML MoS; medio-lateral margin of stability. Bold indicates statistical significance.

The Spearman rank correlation between the total percentage of quarters completed and the ML MoS showed a weak negative correlation (Spearman’s rho = −0.279, *p* = 0.077), indicating a weak relationship between the 2 variables. Although this did not reach statistical significance, the result may suggest a trend whereby participants who completed more quarters on the balance board tended towards a lower ML MoS (*p* < 0.1).

### Energy cost of walking

Mean comfortable walking speed was 1.2 m/s (SD 0.21) and fast walking speed 1.41 m/s (SD 0.28). At CWS the ECw was 4.99 J/kg/m (SD 0.70) and at FWS 4.84 J/kg/m (SD 0.55), which did not significantly differ. Energy expenditure per minute of walking significantly increased when walking at FWS. All spatiotemporal parameters changed significantly, except for step width. Cadence, step length, and stride length increased significantly and percentage double support for the FWS decreased compared with CWS (Table II).

### Relationship between ML MoS and ECw

The ML MoS of the MIL was positively associated with ECw (β = 47.67, 95% CI: 16.87–78.48), as was walking speed (β = 2.54, 95% CI: 0.39–4.70). Walking speed was identified as a significant effect modifier of this relationship (β = −27.07, 95% CI: −49.32; −4.83), indicating that the association between ML MoS and ECw differed according to walking speed. As a significant interaction was present, the main effects should be interpreted in the context of the full model. Walking speed as a dichotomous value (CWS = 0, FWS = 1) was added to the model to check for confounding. This determinant was not a confounder, so it was left out of the final model (Table III).

**Table III:** Estimates of the fixed coefficients with 95% confidence intervals of the linear mixed models predicting energy cost of walking, including the interaction between medio-lateral margins of stability and walking speed

Fixed coefficients	Estimate	95% CI	
		Lower	Upper
Most impaired leg			
Intercept	0.21	–2.94	3.37
Medio-lateral margins of stability MIL (m)	47.67	16.87	78.48
Walking speed (m/s)	2.54	0.39	4.70
Medio-lateral margins of stability MIL* walking speed	–27.07	–49.32	–4.83
Least impaired leg			
Intercept	–0.45	–4.66	3.77
Medio-lateral margins of stability LIL (m)	60.37	16.71	104.02
Walking speed (m/s)	3.91	1.08	6.75
Medio-lateral margins of stability LIL * walking speed	–45.09	–76.05	–14.13
Balance board			
Intercept	6.81	5.54	8.07
Balance board quarters (%)	–0.14	–0.23	–0.05
Walking speed (m/s)	–1.25	–2.35	–0.15
Balance board quarters * walking speed	0.08	0.02	0.15

95% CI: 95% confidence interval; MIL: most impaired leg; LIL: least impaired leg; m: metre; m/s, metres per second.

**Fig. 2:**
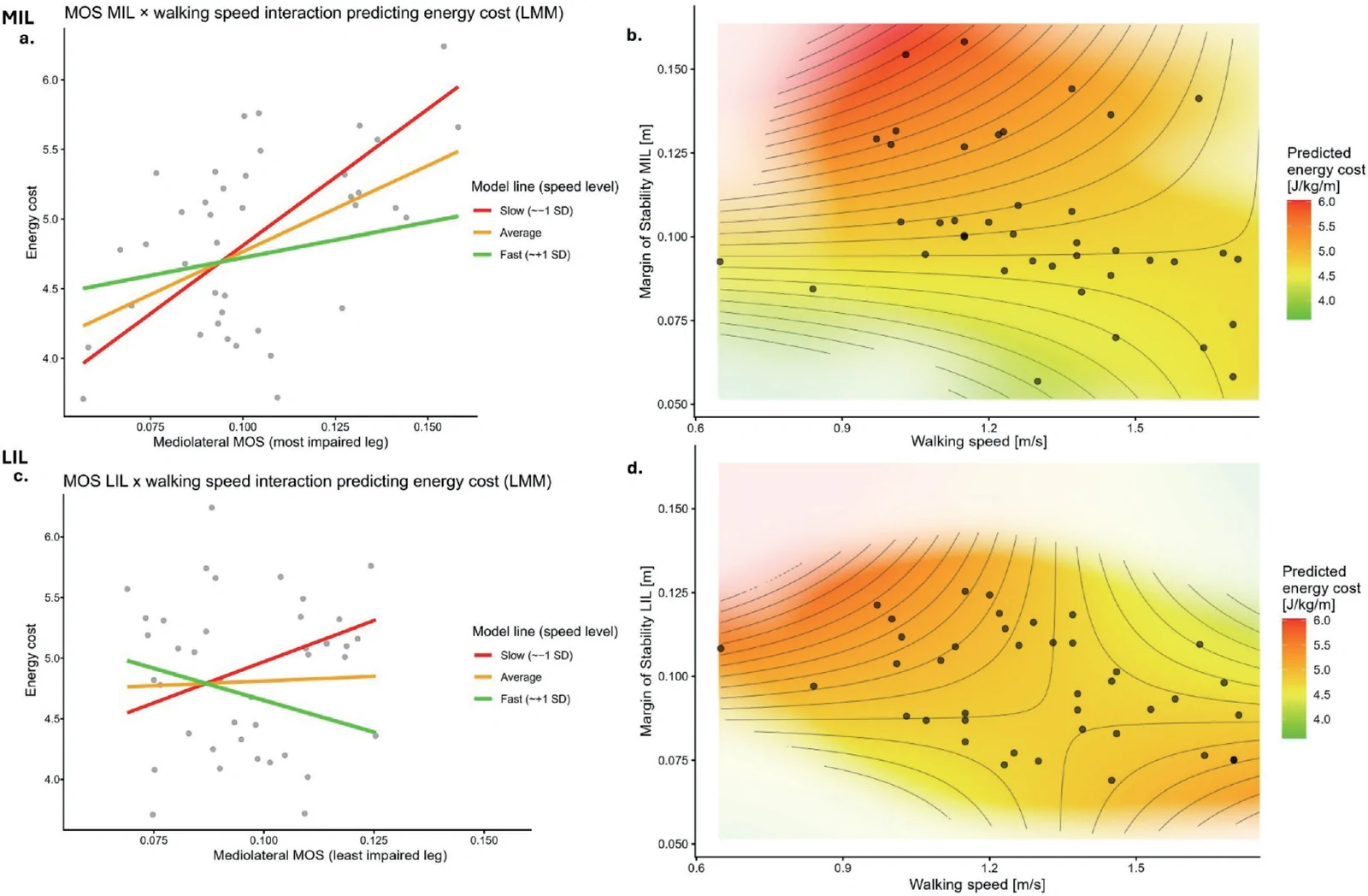
Scatter plots showing the interaction between ML MoS and walking speed in modelling energy cost of walking (ECw) for slow (−1 SD), average and fast (+1 SD) walking speed for most impaired leg (MIL) (a.) and least impaired leg (LIL) (c.). In (b) (MIL) and (d) (LIL) the relationship between walking speed (m/s), mediolateral margin of stability (ML MoS, in cm) and predicted ECw (J/kg/m) are presented, showing how variations in walking speed and ML MoS are associated with changes in ECw. The color gradient reflects modelled ECw, with green indicating lower and red indicating higher ECw. The color scale is constrained to the range of observed data to avoid extrapolation into regions with few or no observations. The contour lines represent equally modelled ECw (J/kg/m) and data points represent individual observations.

The interaction of walking speed within the association between ML MoS and ECw is shown in Fig. 2. In Fig. 2B/D ECw is indicated using a colour gradient, with green indicating the lowest ECw. The contour lines represent equal predicted ECw and show how ECw varied with changes in walking speed and MoS. Higher ECw was associated with greater MoS and lower walking speeds, while walking at a higher walking speed was associated with lower ECw.

The association between ML MoS and ECw for the LIL showed that walking speed was a significant effect modifier in this relationship as well (Table III).

### Relationship between balance board score and ECw

The final model included percentage of balance board quarters completed, walking speed and the interaction term (balance board score × walking speed). Walking speed was identified as a significant effect modifier (β = 0.08, 95% CI: 0.02; 0.15), indicating that the association between balance board performance and ECw differed according to walking speed. As effect modification was established, walking speed was retained in the final model as a moderator (Table III). The model showed that a higher balance board score (i.e., better dynamic balance) was associated with a lower ECw, more so at lower walking speeds.

## DISCUSSION

The primary aim of this study was to investigate the relationship between dynamic balance and ECw, as quantified by the ML MoS and the balance board score, in PwMS. The main findings demonstrate that both ML MoS and balance board score are significant predictors of the energy cost during walking in PwMS and that this relationship is modified by walking speed. At lower walking speeds, an increase in ML MoS is associated with a relatively large increase in ECw, whereas at higher walking speeds, the additional ECw associated with a larger ML MoS becomes smaller. The same was true for lower balance board scores.

The study results support our hypothesis that PwMS with more pronounced balance problems exhibit a higher ECw, especially at slow walking speeds. This is consistent with the limited available literature in MS and other patient groups (11, 25, 26). Kalron et al. showed a significant association between an increase in gait variability, measured as step time variability – an indirect measure of dynamic balance – and a higher ECw in PwMS without a history of falls (11). This relationship between balance and ECw was also seen in healthy persons above 60 years (25), and in patients with a spinal cord injury (26). To our knowledge, no previous study has directly examined the association between balance performance and ECw in PwMS across different walking speeds. However, indirect evidence suggests that walking speed plays an important role in this relationship, as slower walking speeds have been shown to disproportionately increase ECw in PwMS (9), and balance impairments are known to be more pronounced at lower walking speeds in this population (16).

In our study population, self-reported gait disability, measured by the MSWS-12, was minimal to mild, which was also reflected in the average walking speed with speeds comparable to the lower range of normative values for healthy adults, and relatively low EDSS score 3.3 [IQR: 2.0; 3.5]. Higher ECw was associated with greater MoS and lower walking speeds. And although not significant, faster walking seems more energetically efficient in our population, in line with Theunissen et al. who demonstrated that PwMS have an energetically optimal walking speed comparable to healthy subjects but with a higher energetic cost and preferred walking speed below this energetic optimum (9). Moreover, this lower walking speed may not necessarily lead to improved balance, as previous literature showed that at higher walking speeds dynamic stability improved (19). This indicates that other factors contribute to the increased energy cost of walking, such as impaired motor control, decreased push-off power, relative aerobic load, sensory problems, or spasticity (27–29).

The observed association between larger ML MoS, reflecting wider-step compensatory strategies associated with medio-lateral instability, and increased ECw may be explained by compensatory gait strategies adopted by PwMS with balance problems. Such individuals seem to walk at slower speeds for safety reasons and in an attempt to reduce fall risk (14, 15). However, these adaptations are biomechanically less efficient and are associated with an increased ECw (30). Stability-enhancing gait adaptations require more frequent muscle activation and limit the body’s ability to exploit natural, energy-saving movement patterns, thereby increasing ECw (31). Several factors may explain why PwMS do not increase their walking speed to reach a more economical gait: the relative aerobic load of walking in PwMS is already close to the first ventilatory threshold (VT1), the threshold between mild and moderate exercise intensity (29), which may discourage faster walking to avoid unsustainable exertion during prolonged or community walking. Also, compensation strategies for tripping are less effective in PwMS, as movements essential for tripping recovery, such as increase of hip and knee flexion and ankle dorsiflexion, are often impaired (32, 33). Reducing walking speed and a wider ML MoS seem a deliberate psychophysiological adaptation rather than a purely mechanical one. Together, these observations highlight the pivotal role of walking speed in the relationship between dynamic balance and ECw in PwMS; at lower walking speeds, larger ML MoS was associated with a higher energy cost, whereas this association was attenuated at higher speeds, suggesting that interventions focusing solely on balance may be insufficient if walking speed is not simultaneously addressed. Furthermore, there exists a moderate correlation between EDSS and walking speed in our sample (*r* = –0.44, *p* = 0.004), which implies that the moderating role of walking speed should be interpreted with appropriate caution, as walking speed may partly reflect between-subject differences in neurological impairment rather than the effect of speed per se. Nevertheless, additional analyses in which EDSS replaced walking speed as a moderator yielded non-significant interactions across all balance measures (Tables SI–SIII), suggesting that walking speed captures clinically relevant variance in the balance–ECw relationship beyond what disease severity alone explains.

Speed-intensive gait training has been shown to simultaneously improve walking speed, endurance, and balance in PwMS without increasing fatigue (34), and may therefore represent a promising approach to reduce ECw in this population. Although the causal direction between walking speed and stability was not directly investigated in this study, our results are consistent with the interpretation that walking at slower speeds as a compensatory strategy to enhance stability may come at the expense of decreased walking efficiency, underscoring the importance of targeting both balance and walking speed simultaneously in the rehabilitation of PwMS. As gait analysis procedures are time consuming and only available in certain centres, the balance board test might offer an easy-to-use alternative as a dynamic balance measurement (12).

### Strengths and limitations

Using overground CWS to set treadmill walking speed ensures that participants walk at a speed representative of their habitual gait, rather than a lower treadmill-adapted speed. This improves ecological validity. However, at the same time our findings are derived from treadmill walking, and it remains unclear how well these results generalize to real-world overground walking, where additional perturbations, terrain variation, and dual-task demands are prevalent (35, 36). Another strength is that balance and ECw, as quantified by the ML MoS, were measured concurrently during the same walking trial. A clear advantage of treadmill walking is that it enables calculation of the ML MoS over numerous steps in a relatively short time span and at a fixed continuous walking speed. This is harder for walking in an overground lab, in which turning during walking trials is most often inevitable.

There are some limitations that should be considered. First, it can be questioned whether treadmill walking can be generalized to community walking. Community walking might impact balance differently than treadmill walking, as in the community perturbations and dual-tasking situations are prevalent. However, from our findings in a controlled lab environment, it can be expected that balance problems can lead to more disturbances in community walking. Second, while walking on a split-belt treadmill, individuals walk with wider steps than during overground walking. This limits the possibilities to utilize lateral foot placement for balance modifications and may mask the role of foot placement compared with single-belt treadmill or overground walking (37). Patients with mild balance problems might walk with wider steps than expected based on their balance problems, while patients with more pronounced balance problems might not increase their step widths further than expected. Therefore, the ML MoS value of the patients with mild balance problems might be closer to the ML MoS value of patients with more pronounced balance problems than would be the case on a single-belt treadmill, which could have reduced the strength of the observed relationship between ML MoS and ECw.

### Conclusion

The association between dynamic balance, measured as ML MoS, and ECw in PwMS is dependent on walking speed. Specifically, poorer dynamic balance was associated with higher ECw predominantly at lower walking speeds, while this association was attenuated at higher walking speeds. PwMS who walk slower and with a larger ML MoS showed higher ECw, compounding their energetic burden. These findings highlight the need for rehabilitation strategies that simultaneously address both dynamic balance and walking speed, as targeting either in isolation may be insufficient to optimize walking efficiency in PwMS.

## Supplementary Material


