# Supplementary material for: THE COST OF IMBALANCE: HOW BALANCE PROBLEMS DRIVE ENERGY COST OF WALKING IN PERSONS WITH MULTIPLE SCLEROSIS
Source: J Rehabil Med. 2026 Sep 18;58:45814. doi: 10.2340/jrm.v58.45814 (PMC13596029; doi:10.2340/jrm.v58.45814)
Supplement: Supplementary file 1 [file JRM-58-45814-s1.pdf]

Supplementary Table I. Energy cost of walking models for the most impaired leg

Fixed Coefficients	Estimate	95%CI	
		lower	upper
**Most impaired leg – speed continuous**			
Intercept	0.21	-2.94	3.37
Medio-lateral margins of stability MIL (m)	47.67	**16.87**	**78.48**
Walking speed (m/s)	2.54	**0.39**	**4.70**
Medio-lateral margins of stability MIL* walking speed	-27.07	**-49.32**	**-4.83**
**Most impaired leg - EDSS**			
Intercept	4.98	**0.47**	**9.48**
Medio-lateral margins of stability MIL (m)	-4.08	-51.05	42.89
EDSS	-0.39	-1.90	1.12
Medio-lateral margins of stability MIL * EDSS	4.61	-9.89	19.11
**Most impaired leg - speed categorical**			
Intercept	3.22	**2.13**	**4.30**
Medio-lateral margins of stability MIL (m)	16.03	**5.97**	**26.10**
Walking condition (CWS =0 FWS=1)	0.71	-0.05	1.48
Medio-lateral margins of stability MIL * walking condition	-6.96	-14.16	0.24

Abbreviations: 95%CI, 95% confidence interval; MIL, most impaired leg; EDSS, Expanded Disability Status Scale; CWS, comfortable walking speed; FWS, fast walking speed

Supplementary Table II. Energy cost of walking models for the least impaired leg

Fixed Coefficients	Estimate	95%CI	
		lower	upper
**Least impaired leg – speed continuous**			
Intercept	-0.45	-4.66	3.77
Medio-lateral margins of stability LIL (m)	60.37	**16.71**	**104.02**
Walking speed (m/s)	3.91	**1.08**	**6.75**
Medio-lateral margins of stability LIL * walking speed	-45.09	**-76.05**	**-14.13**
**Least impaired leg EDSS**			
Intercept	1.94	**0.48**	**9.49**
Medio-lateral margins of stability LIL (m)	21.50	-51.05	42.90
EDSS	1.12	-1.90	1.12
Medio-lateral margins of stability LIL * EDSS	-8.40	-9.89	19.11
**Least impaired leg - speed categorical**			
Intercept	4.01	**2.22**	**5.80**
Medio-lateral margins of stability LIL (m)	9.07	-8.94	27.09
Walking condition (CWS =0; FWS=1)	0.86	-0.30	2.03
Medio-lateral margins of stability LIL * walking condition	-9.11	-20.99	2.76

Abbreviations: 95%CI, 95% confidence interval; LIL, least impaired leg; EDSS, Expanded Disability Status Scale; CWS, comfortable walking speed; FWS, fast walking speed

Supplementary Table III. Energy cost of walking models for Balance board

**Fixed Coefficients**	**Estimate**	**95%CI**	
		**lower**	**upper**
**Balance board – speed continuous**			
Intercept	6.81	**5.54**	**8.07**
Balance board quarters (%)	-0.14	**-0.23**	**-0.05**
Walking speed (m/s)	-1.25	**-2.35**	**-0.15**
Balance board quarters * walking speed	0.08	**0.02**	**0.15**
**Balance board - EDSS**			
Intercept	5.87	**3.63**	**8.11**
Balance board quarters (%)	-0.02	-0.05	0.004
EDSS	-0.21	-0.87	0.45
Balance board quarters * EDSS	0.01	-0.003	0.01
**Balance board - speed categorical**			
Intercept	5.46	**4.99**	**5.93**
Balance board quarters (%)	-0.01	-0.02	0.003
Walking condition (CWS =0; FWS=1)	-0.42	**-0.68**	**-0.17**
Balance board quarters * Walking condition	0.01	**0.003**	**0.01**

Abbreviations: 95%CI, 95% confidence interval; EDSS, Expanded Disability Status Scale; CWS, comfortable walking speed; FWS, fast walking speed
